# Ultrafast Laser Fabrication of Functional Biochips: New Avenues for Exploring 3D Micro- and Nano-Environments

**DOI:** 10.3390/mi8020040

**Published:** 2017-01-28

**Authors:** Felix Sima, Jian Xu, Dong Wu, Koji Sugioka

**Affiliations:** 1 RIKEN Center for Advanced Photonics, Wako, Saitama 351-0198, Japan; jxu@riken.jp; 2 Center for Advanced Laser Technologies (CETAL), National Institute for Laser, Plasma and Radiation Physics (INFLPR), Atomistilor 409, 0077125 Magurele, Romania; 3 University of Science and Technology of China, Hefei 230026, China; dongwu@ustc.edu.cn

**Keywords:** femtosecond laser assisted etching, two photon polymerization, ship-in-a-bottle laser processing, glasses, polymers, biochips, biomimetic environments

## Abstract

Lab-on-a-chip biological platforms have been intensively developed during the last decade since emerging technologies have offered possibilities to manufacture reliable devices with increased spatial resolution and 3D configurations. These biochips permit testing chemical reactions with nanoliter volumes, enhanced sensitivity in analysis and reduced consumption of reagents. Due to the high peak intensity that allows multiphoton absorption, ultrafast lasers can induce local modifications inside transparent materials with high precision at micro- and nanoscale. Subtractive manufacturing based on laser internal modification followed by wet chemical etching can directly fabricate 3D micro-channels in glass materials. On the other hand, additive laser manufacturing by two-photon polymerization of photoresists can grow 3D polymeric micro- and nanostructures with specific properties for biomedical use. Both transparent materials are ideal candidates for biochips that allow exploring phenomena at cellular levels while their processing with a nanoscale resolution represents an excellent opportunity to get more insights on biological aspects. We will review herein the laser fabrication of transparent microfluidic and optofluidic devices for biochip applications and will address challenges associated with their potential. In particular, integrated micro- and optofluidic systems will be presented with emphasis on the functionality for biological applications. It will be shown that ultrafast laser processing is not only an instrument that can tailor appropriate 3D environments to study living microorganisms and to improve cell detection or sorting but also a tool to fabricate appropriate biomimetic structures for complex cellular analyses. New advances open now the avenue to construct miniaturized organs of desired shapes and configurations with the goal to reproduce life processes and bypass in vivo animal or human testing.

## 1. Introduction

Ultrafast lasers are generating ultrashort pulses of light, with durations less than a few picoseconds, typically in the femtosecond (fs) regime. This means that the energy deposition on a material occurs at a timescale shorter than electron–phonon coupling processes, reducing formation of heat-affected zone for high-quality micro and nano fabrication [1]. Additionally, the extremely high peak power (P_peak_ = E/τ, E-pulse energy, τ-pulse duration) of ultrafast lasers can induce nonlinear absorption processes in materials which do not absorb the laser wavelength, and thus allows processing inside of transparent materials [2,3].

The first micromachining trials and demonstration with ultrafast lasers were performed on silica [4] while formation of submicron holes on metal surfaces was performed in the sequel [5]. The fundamental aspects on mechanisms were evaluated in a systematic study of ultrafast laser interaction with various transparent dielectric materials such as glasses, crystals and polymers [6]. Later, ultrafast laser processing at micro- and nanoscale was applied to remove material or modify properties for both opaque and transparent materials. Versatile capability of ultrafast lasers in terms of both the kinds of materials processed and the types of processing extends applications to broad collections including simple ablation, nanostructring, 3D printing, and waveguide and microchannel fabrication. For transparent glass materials including silica, borate, soda lime silicate, and fluorozirconate, the ultrafast laser modification based on multiphoton interactions induces a permanent phase or structural change due to a densification generated by either pressure wave or fast heating-cooling processes [7,8,9]. Eventually, defects, nonbridging oxygen hole centers, and peroxy radicals are formed at the focal volume, facilitating the fabrication of 3D optical circuits in bulk glasses with laser direct writing [10]. Microchannels with 3D geometries were also fabricated in photosensitive glass by ultrafast laser direct writing followed by wet chemical etching with hydrofluoric acid (HF). It was found that the thermal treatment after the laser direct writing gave faster etching rate to the laser exposed regions as compared with the unexposed regions, resulting in formation of channels inside glass for fabrication of micro-total-analysis systems [11]. A similar process was also applicable to fused silica, although the higher etch rate can be achieved immediately after laser irradiation without any thermal treatment [12,13]. Polymers are also available for fabrication of both 3D optical circuits [14] and 3D microchannels [15,16]. They exhibit some complementary advantages over glasses such as low cost, dopant availability, elasticity, and processing at an intensity reduced with one order of magnitude. However, due to the higher transmission losses and low etching selectivity, fewer studies were reported for fabrication of waveguides or holes in poly(methyl-ethacrylate (PMMA) [17,18], gratings or micro-lenses in polydimethylsiloxane (PDMS) [19,20], and microchannels [15]. On the other hand, ultrafast laser induced photopolymerization of resins can directly create 3D micro- and nanostructures. This process known as two photon polymerization (TPP) is an alternative to conventional soft lithography methods with the advantages involving no requirement of any mask or supplementary processing phases and capability of fabricating 3D polymeric structures at nanoscale with high degree of complexity [21,22,23]. This significantly shifts the bio-device size from micro to nano scale [24]. There are several review articles available on ultrafast laser processing of glass, polymers and photoresist for optofluidic, photonic and biochip applications [3,25,26,27,28,29,30,31,32] including future perspectives of combination of biomimetics and laser technologies [33].

In this review, we will highlight on the ultrafast laser micromachining of transparent materials, in particular glasses and photoresists, for the fabrication of functional biochips. The miniaturized setups created inside glass permit the use of only small quantities of reagents for biological tests. Due to the flexibility of tailoring 3D geometries, functional components can be fabricated in microfluidic devices for specific biological assays. The glass is a suitable material for biochip platform due to transparency, robustness, easy manipulation and high portability, while polymeric structures provide the sub-micron features and elasticity necessary for specific biomedical applications.

## 2. Surface Processing of Transparent Materials for Biochip Application

Glasses are excellent materials for lab-on-a-chip devices, however the cost in terms of both material itself and production of devices is rather high [34,35]. Microfluidic biochip devices fabricated in plastics appear very attractive since they are cost-effective and involves simplified manufacturing process as compared with glass [36]. Many technologies have been developed with the aim of creating a functional device capable to provide accurate analysis and low cost, which involve molding, hot embossing and thermoforming methods depending on processed materials and specific applications [37]. In contrast, laser micromachining was found a viable method to process transparent material due to its unique features; (i) fast prototyping technology; (ii) flexible engineering of complex shapes; and (iii) diversity in terms of kinds of materials processed from glasses to polymers and biomaterials [38]. In particular, laser is a reliable tool to create channels on the material surfaces for transporting samples and reagents at specific places. The fluid control can be archived by microvalves or micropumps also fabricated by laser on the same substrates. As a consequence biochips integrated with a variety of functional biophotonic components can be fabricated by laser [24]. Laser based micro-and nanofabrication techniques such as direct write methods, matrix assisted pulsed laser evaporation, laser-induced forward transfer, and near and far field processing were summarized in several reviews with critical comparison to conventional techniques [24,39,40]. Some very specific applications were also proposed to functionalize the material by laser: collagen microtracks for cancer cell invasion [41], agarose microchannels for neural cell research [42] and hyaluronic acid patterns for tailoring adhesion and proliferation of endothelial cells [43]. However, simple surface processing does not satisfy needs for complex microfluidic applications and is rather difficult to create biomimetic environments as they usually appear in 3D spaces. Consequently, ultrafast laser 3D processing emerged as alternative to the other laser based methods for the fabrication of true 3D biochips with specific functionalities and biomimetic characteristics.

## 3. Methods Specification

Femtosecond laser assisted etching (FLAE) applied to glasses is classified as a subtractive process since the irradiated zones are chemically removed by successive HF etching. FLAE was applied both to photosensitive glasses like Foturan and to fused silica due to modification of chemical properties besides the refractive index [12,13,44]. The method can also be called Femtosecond Laser Irradiation followed by Chemical Etching (FLICE) [27,45,46]. 

Each glass provides their own strength and weakness in terms of biochip applications. Fused silica exhibits better optical performances such as broader transmission range and lower autofluorescence. On the other hand, Foturan allows fabricating larger scale of complex microfluidic structures due to more efficient processing parameters such as higher writing speed and higher etch rate. More importantly, Foturan can be thermally treated after the etching in order to obtain smooth surface with nanoscale roughness [26]. As a consequence of smooth surface profiles of Foturan by FLAE, one can integrate microoptical components easier into 3D microfluidic structures to realize functional optofluidic systems.

**Figure 1:**
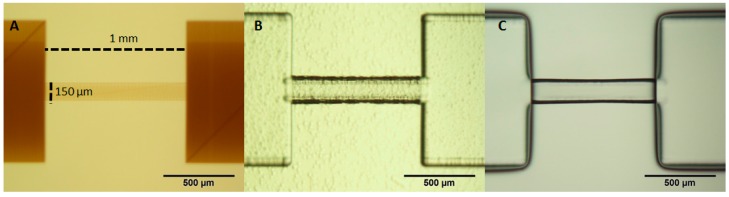
Microscopic images of Foturan glass exposed to ultrafast laser irradiation followed by a first annealing treatment (**A**); 45 min chemical etching in 10% HF (**B**); and smoothening by additional annealing treatment (**C**).

Selective etching mechanism of Foturan is completely different from that of fused silica. It is considered that enhanced etching selectivity for fused silica is originated from the weakened chemical bonds produced by densification. In contrast, the modification of Foturan relies on photochemical process due to its unique composition. Specifically, Foturan is a lithium-aluminosilicate glass doped with trace amounts of silver and cerium ions in order to print photographic images when exposed to UV radiation followed by thermal treatment [47]. It was found that ultrafast laser irradiation generates free electrons in Foturan by multiphoton absorption [48] that are captured by silver ions to precipitate silver atoms. The laser irradiation is followed by an annealing treatment in which precipitated Ag atoms are clustering to form Ag nanoclusters. These act as nuclei to grow a crystalline phase of lithium metasilicate within laser exposed regions. This modification is visibly observed inside photosensitive glass due to color change (Figure 1A). The subsequent wet chemical etching using diluted HF selectively etches the modified regions, in which the time is critical parameter to get uniform channels with similar width, in particular over long lengths (Figure 1B). A post-thermal treatment is further employed to decrease the roughness generated by the etching to the nanometer range (Figure 1C) [49]. This additional annealing performed slightly above the glass softening temperature offers highly transparent samples for clear optical integration. One can get microfluidic platforms with the scale-down and scale-up characteristics for fabrication of functional microfluidic devices.

Two-photon polymerization (TPP) using photo-curable resin or negative-tone photoresists is an additive process that can tailor shape and size of the solidified polymer volume element (voxel). A photoinitiator mixed in the resin develops free radicals when irradiated with UV light, which lead to polymerization of monomers in the resin. Alternatively, the free radicals can be formed when the photoinitiator simultaneously absorbs at least two photons when irradiating with ultrafast laser. TPP takes place only at the focal point of the laser beam by adjusting the laser energy unlike single-photon polymerization using UV light which occurs along the entire exposed pattern. Thus, 3D structures are localized and developed by translating the focal point of ultrafast laser within the resin. The capability of controlling the feature sizes in the micro- and nanoscale and high flexibility arising from laser direct writing scheme has pushed TPP to become a well-known technology for fabricating micro and nanodevices with complex designs.

**Figure 2:**
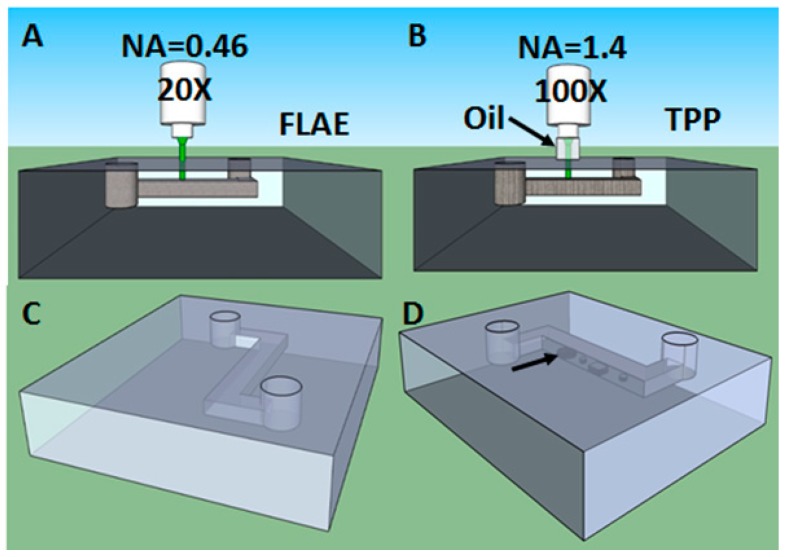
Schematic of hybrid femtosecond laser assisted etching followed by two-photon polymerization (FLAE-TPP) process for ship-in-a-bottle polymer integration: FLAE of Foturan glass (**A**); TPP of SU-8 photoresist inside microchannel (**B**); Z-shape microchannel fabricated by FLAE (**C**); and polymeric patterns (indicated by black arrow) developed by TPP inside microchannel (**D**).

Combining advantages and compensating drawbacks of both subtractive and additive processes, a new concept of hybrid 3D processing was proposed to create 3D micro- and nanostructures with more complicated shapes and increased functionalities. Specifically, after the 3D channel fabrication by FLAE (Figure 2A,C), the fabricated channel is filled with an epoxy negative-tone photoresist with good mechanical, chemical and biological properties. TPP process is then carried out in order to create polymeric structures inside the channels (Figure 2B,D). In order to increase the resolution, an oil immersion objective lens with higher numerical aperture is used for TPP. The hybrid process allows lowering the size inside the glass microchannels due to much higher fabrication resolution of TPP than FLAE. It offers in the same time to maintain the transparency for optical interrogation as well as robustness for fabrication of a concrete device.

We summarize the ultrafast laser 3D processing methods described above with specific advantages and drawbacks in terms of biochip fabrication in Table 1.

## 4. Subtractive 3D Glass Micro Fabrication

**Figure 3:**
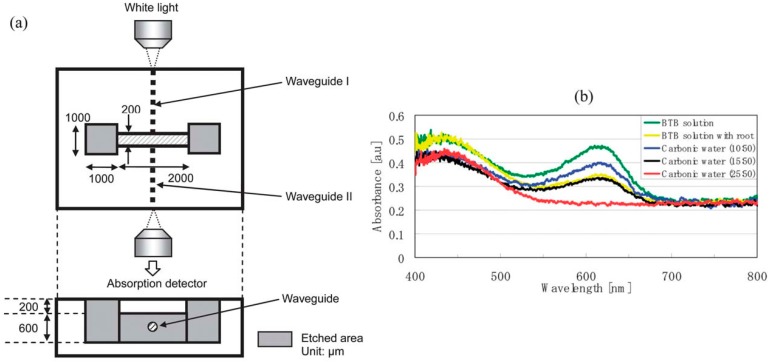
(**a**) Schematic of the microfluidic chip integrated with optical waveguides. White light for absorption measurements is transmitted by waveguide I to cross the microfluidic channel filled with liquid samples and is then coupled into optical waveguide II to be introduced to the detector; (**b**) Optical absorption spectra of liquid samples filling the microfluidic channel: water containing an aqueous bromothymol blue solution (green line), with a seedling root (yellow line), and with carbonic water with different CO_2_ concentrations [50].

Different structures of 3D microfluidic channels were successfully produced by FLAE in Foturan glass. In particular, optical waveguides and filters were integrated by ultrafast laser processing with the microfluidic structures for fabrication of optofluidics [50]. This allowed understanding the gliding mechanism of *Phormidium*, which is a genus of filamentous cyanobacteria and useful to accelerate growth of vegetables. To investigate the attraction mechanism that directs *Phormidium* to glide toward a seedling root, a T-shaped microfluidic channel formed in Foturan glass was fabricated, with three reservoirs at its ends. When *Phormidium* was introduced into one reservoir and a seedling root into another, the *Phormidium* always glided toward the seedling root rather than toward a third empty reservoir. On the other hand, when the third reservoir was filled with carbonic water, the direction that *Phormidium* glided depended on the carbonic water concentration. Additionally, at a critical CO_2_ concentration, the cyanobacterium glided neither toward the seedling root nor toward the carbonic water, indicating that CO_2_ secreted by respiration of the root is a possible attractant. To confirm this hypothesis and determine the quantity of CO_2_ secreted by the seedling roots, an optofluidic system similar to those used to detect and manipulate single cells was fabricated in Foturan. Figure 3a shows a schematic illustration of the fabricated optofluidic system. After fabricating a simple straight microfluidic channel in the glass, optical waveguides were written that intersect the center of the microfluidic channel. The microfluidic channel was filled with water containing a pH indicator (bromothymol blue (BTB) solution) and white light was coupled to the entrance facet of waveguide I by an objective lens. The white light transmitted by waveguide I passed through the microfluidic channel, which was filled with a liquid sample, and was then coupled into optical waveguide II. The light transmitted by waveguide II was coupled into a spectrometer by another objective lens to obtain the spectrum of white light absorbed by the sample. The green line in Figure 3b indicates the absorption spectrum of the water containing BTB solution. It has a large absorption peak at a wavelength of about 620 nm. The intensity of this peak decreases with increasing CO_2_ concentration in the water due to the change in the pH. The spectrum of water containing the seedling root (yellow line) is comparable to that of 50 mL water mixed with 15 mL CO_2_ (black line). This result implies that the CO_2_ concentration generated by the root’s respiration is comparable to that of the carbonic water (15 mL CO_2_: 50 mL H_2_O) used in this experiment. Interestingly, this CO_2_ concentration is equal to the critical concentration at which *Phormidium* did not glide toward either the seedling root or the carbonic water in the T-shaped microfluidic channel, confirming that CO_2_ is the sole attractant for the gliding *Phormidium*. Additionally, it was found that CO_2_ secreted from the seedling root attracts *Phormidium* in the presence of light. Integration of optical filters further revealed the light intensity necessary for gliding. *Phormidium* starts to glide to a seedling root when white light intensity is above a threshold estimated at 1530 lx. By using band-pass filters it was determined that red light only (640–700 nm) promotes *Phormidium* gliding. Based on the obtained results, one can suppose that new methods for accelerating vegetable seedling growth can be further developed.

Another design of optofluidic devices was proposed to perform highly sensitive biochemical liquid assays. It consisted of a microfluidic channel with polymer coated internal walls and an optical waveguide embedded in Foturan glass structured by ultrafast laser [51]. The polymer coating allowed the probe light introduced by the optical waveguide to propagate along microfluidic channel, since the refractive index of coated polymer was smaller than the liquid samples. Bovine serum albumin concentrations down to 7.5 mM as well as 200 nM glucose-D were analyzed by absorption measurements.

The integration of waveguides and some other micro-optical components in glass microfluidic structures using the same processing laser is beneficial for optofluidic applications. Due to post-annealing treatments required for smoothening the etched surfaces, the waveguides are generally written in samples just after the fabrication of the micro-optical and microfluidic components by FLAE. This increases the difficulty of the fabrication process in terms of alignment of each component. Nevertheless, such integration definitely enhances device functionality. 

Integrated biochips with optical components have been successfully demonstrated for highly sensitive analysis of liquid samples [50,51,52,53] and determination of functions of microorganisms [54,55]. A concrete example is an integrated optofluidic sensor that is capable of performing both absorption and fluorescence spectroscopic detection of liquid samples [52]. More specifically, a long optical waveguide is connected to a microfluidic reservoir. The waveguide was used to transfer either the fluorescence excitation light from a laser or a broadband beam from a white lamp for absorbance measurements of liquid samples confined in the microreservoir. To avoid divergence of optical signals, two microspherical lenses structured by FLAE were integrated at the side of and behind the microreservoir. Such integration highly increased the efficiency of both the fluorescence emission and transmission light spectroscopy.

**Figure 4:**
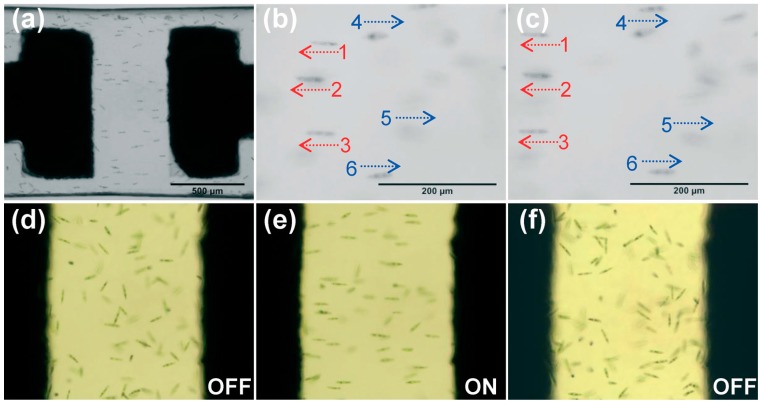
Electroorientation of Euglena in microfluidic channel: (**a**) Overview of oriented *Euglena* cells between a pair of microelectrodes (black parts) in a channel when applying an AC voltage. (**b**,**c**) Oriented *Euglena* cells swimming bidirectionally at different times. Movement of *Euglena* in the microchannel: (**d**) before applying the electric field; (**e**) with electric field; and (**f**) when the electric field was turned off. Reproduced from [56].

To increase functionalities of biochip devices, a space-selective metallization of microfluidic structures using ultrafast laser direct-write ablation followed by electroless metal plating was proposed [56]. The hybrid method which includes FLAE followed by fs laser ablation and electroless metal plating allows flexible coating with metal films into desired locations inside microfluidic structures. This technique was then applied to integrate two opposed microelectrodes in a straight microfluidic channel (Figure 4). The fabricated electrofluidic device allowed the orientation of asymmetric biological samples, such as *Euglena gracilis*, an aquatic microorganism.

In addition, a new method for the fabrication of vertical electrodes on the sidewalls of 3D glass microfluidic channels was developed. By using water-assisted ultrafast laser ablation followed by electroless plating one can obtain high-quality metallic coatings onto ablated patterns [57]. Then, sidewall metal patterning in 3D microfluidic structures were achieved, enabling continuous metal wiring from the inside to the outside of microfluidic structures. Further, 3D glass microfluidic structures monolithically integrated with vertical electrodes were proposed to be used for movement control of Caenorhabditis elegans (*C. elegans*), a free-living transparent nematode (worm), in channels based on electrotaxis. Importantly, it should be noted that the selective metallization by this technique is available only for Foturan glass and does not work for fused silica.

Similar subtractive process is also available for fused silica as described above. Long channels with high aspect ratios were fabricated in fused silica by chemical etching using KOH instead of HF which provided a better selectivity between laser exposed and unexposed zones, although the etching rate is extremely low [58]. 

**Figure 5:**
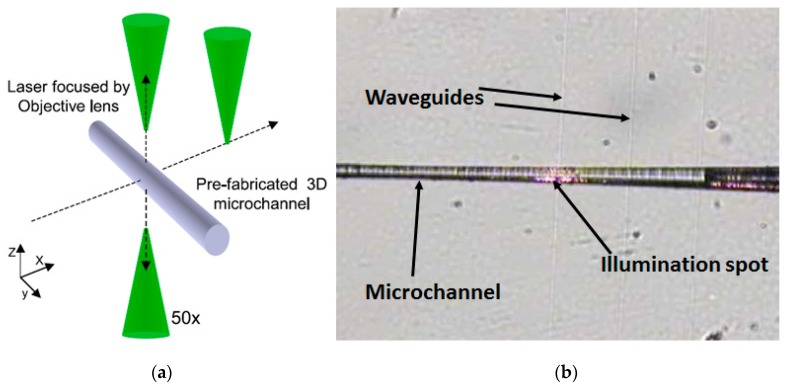
(**a**) Schematic illustration of laser direct writing scheme for flexible integration of optical waveguides; and (**b**) optical microscope image of microchannel integrated with optical waveguides: A He-Ne laser was coupled to one of the waveguides and shows local illumination inside microchannel. Reproduced with permission from [60].

By using the same ultrafast laser it was possible to fabricate both microfluidic channels in fused silica and high quality optical waveguides on the same substrate [59]. The integrated device was proposed for a new class of biophotonic sensors. Further, a fused silica based optofluidic device with integrated waveguides fabricated by ultrafast laser was used for flow tests with fluorescent particles and red blood cells (RBCs) in a 3D configuration [60]. Flexible scheme of ultrafast laser direct writing allows us to integrate both waveguides perpendicular and parallel to the top surface of glass chip as shown in Figure 5a. Figure 5b shows an optical microscope image of combined optical waveguides crossing the pre-fabricated microchannels, by which a He-Ne laser (632.8 nm) beam was successfully coupled through one of waveguides to illuminate locally the microchannel. The channel-waveguide assembling permitted the laser-induced cell processing inside the microchannels and RBCs detection via transmission and fluorescence probing.

**Figure 6:**
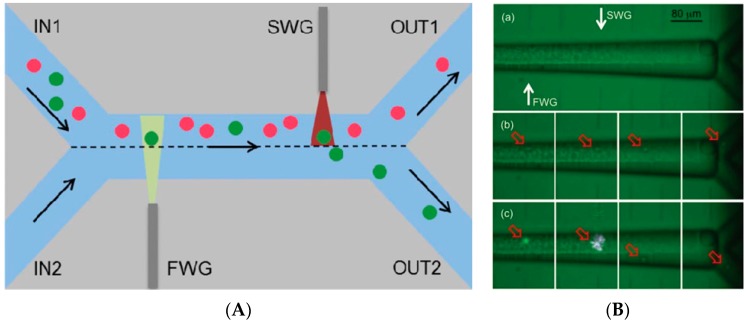
(**A**) Schematic and cell sorting principle where green particles are fluorescent: FWG: “fluorescence waveguide” excitation of cells fluorescence; SWG: “sorting waveguide” used for optical force in sorting process. (**B**) (**a**) Image during operation; (**b**) sequence of frames during fluorescence activation with non-fluorescent cells; and (**c**) with fluorescent cells. Reproduced with permission from [63].

A fused silica based optofluidic monolithic chip consisting of microchannels with a square cross-section geometry and waveguides was fabricated by ultrafast laser for optical trapping and stretching of single cells [61]. Such devices were proposed as microscope platforms for complex cellular investigations. Other configurations were further developed and proposed for real-time sorting optofluidic devices which proved their performance by extracting metastatic cells from a heterogeneous cell mixture [62]. Similar type of optofluidic system was utilized for cell sorting based on optical forces combined with fluorescence detection of the cells [63]. Figure 6A illustrates the principle of cell sorting using this scheme. Two input channels (INs) are merged into a single straight channel where fluorescence detection and sorting are conducted to separate the cells into two output channels (OUTs). The sample liquid containing cells and a buffer solution are introduced into IN1 and IN2, respectively. Appropriate control of the fluid flow rates induces laminar flow in the single straight channel, so that the entire sample with cells is exhausted to OUT1. The application of optical forces pushes cells into the buffer solution side, where the cells are collected in OUT2. Sorting can be automatically performed based on the fluorescence detection of cells, where a fluorescence laser beam is directed by a fluorescence waveguide (FWG) to the microchannel, which illuminates the entire height of the microchannel to detect all cells flowing in the microchannel. The specific fluorescence signal can be detected when the target cells pass through a region in front of the FWG. Detection of the fluorescence signal automatically switches on the optical force laser beam, which is guided to the microchannel by the sorting waveguide (SWG), after a moderate delay time to synchronize with the passage of the detected cells in front of the SWG. The target cells are then pushed into the buffer solution side and eventually sorted to OUT2. Figure 6B shows optical microscope images of the optofluidics demonstrating the cell sorting. The optical force laser beam was kept switched off and the cells continued to flow and be exhausted at OUT1 (Figure 6B-b). In contrast, when a fluorescent cell was illuminated, a fluorescence signal was detected. After an appropriate delay time, the optical force laser beam was switched on to push the cell into the buffer solution side to sort and exhaust to OUT2, as shown in Figure 6B-c.

One could mention that an inherent issue is the limited choice of glass types for FLAE. Foturan and fused silica only are appropriate for FLAE, since the other glasses do not offer sufficiently high selectivity in chemical etching at the locally modified regions [26]. A more important drawback is related to the resolution of the fabricated structures which ranges from a few to tens of micrometers due to wet chemical etching process. On the contrary, TPP described in the next section can achieve higher resolutions of the developed patterns, with sub-100 nm feature size [64].

## 5. Additive Two-Photon Polymerization

There are a broad variety of photoresist materials available for TPP. They exhibit biocompatibility as well as different biodegradability rates, elasticity and porosity. These properties are important factors for biological applications.

The devices first fabricated by TPP were 3D photonic crystals which showed excellent photonic bandgap [65]. Later, filtering structures were integrated in the open microfluidic structure by TPP [66]. Further, integration of movable micro-mechanical and optical components such as a functional microvalve and a microlense in a microfluidic channel was demonstrated.

**Figure 7:**
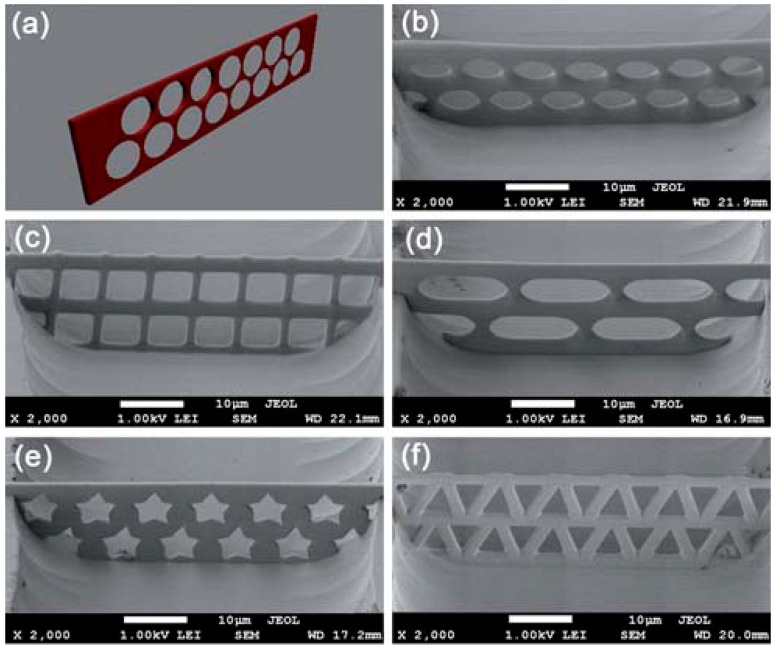
Polymeric structures grown by TPP inside open channel. (**a**) Design of 3D structures with high aspect ratio wall and pores in it with different shapes: round (**b**); square (**c**); round-end rectangle (**d**); pentagrams (**e**); and triangle (**f**). High resolution and high aspect ratio proved the fabrication of complex 3D structures by TPP. Reproduced with permission from [67].

There are, however, some issues related to the stability of photopolymerized structures, especially when they are fabricated with the highest resolution (finer features) for applications such as micro to nano-photonics and microfluidics. The stability problems are due to polymer shrinkages, deformations and collapsing after developing. In order to avoid these issues in biochip applications, in most cases, TPP was used to fabricate 3D microstructures in open glass microfluidic channels prepared by other techniques in advance (Figure 7) [67].

**Figure 8:**
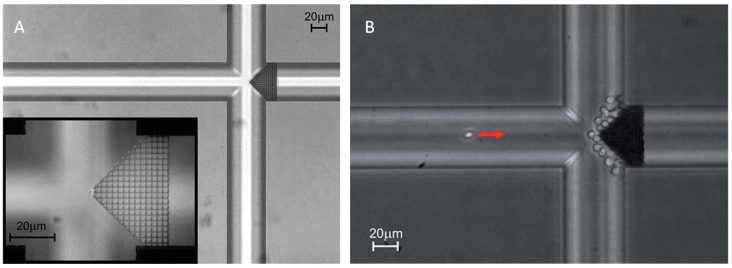
(**A**) Image of the filter inside the microfluidic chip between two channels. Inset shows the filter at a higher magnification. (**B**) Blood cells filtered from diluted blood. Reproduced with permission from [68].

Other studies by TPP were devoted to the integration of a 3D filter with micro-pores in a commercial closed microfluidic chip [68]. With a specific configuration, the filter was placed at the intersection between channels (Figure 8A). Without any clogging for 25 min, filtering tests with a suspension of 3 µm polystyrene spheres in a Rhodamine 6G solution demonstrated that the fluorescent molecules were successfully passed through the filter while the all spheres were captured by the filter. The device functionality was validated using blood cells (Figure 8B).

The integration of polymeric structures inside channels offers a concrete functionality of the whole device in addition to stability. Thus, TPP prototyping becomes a strong method for functionalization and integration of polymeric components with glass microfluidics for the development of lab-on-a-chip devices, which opens new avenues to fabricate novel biochips devices.

On the other hand, 3D scaffolds mimicking features for organisms and cells can be constructed with controlled pore size and desired geometry [69]. This particular feature is important since 3D environments can offer the surrounding support for cells similar with in vivo conditions and could be an alternative to animal testing. Many studies with different biomimetic architectures and different cell lines provided specific cellular responses to 3D constructs [53,70,71,72,73,74,75,76,77,78,79]. Some resists were proposed for measuring the cell force. In particular, Ormocomp® elastic beams (Micro Resist Technology GmbH, Berlin, Germany) were constructed by TPP for the measurement of contraction forces in case of cardiomyocytes [80]. In another study, cells cultured on complex scaffolds developed by TPP enhanced their adhesion sites only on the functionalized area of Ormocomp^®^, so that cellular adhesion control in a 3D environment was proposed [75]. Besides the cellular adhesion, proliferation, differentiation, and cell migration are important phenomena to be monitored for investigations on the regulation of the inflammatory response and formation of tumor metastasis [81]. For example, the evolution of cancer malignancy could be explored to know how the tumor cells can invade other tissues through invasiveness potential. The cells are migrating in a confined 3D environment in response to various physical and chemical stresses. This investigation is attracting a great interest in utilization of 3D biomimetic systems that can be alternative to in vivo models. Such 3D biomimetic systems can potentiate high-throughput analyses on the cell migration mechanisms. Due to flexibility of the process, TPP can create complex 3D matrix structures with controlled pore sizes and desired shapes to mimic the organism. It was actually demonstrated that a 3D microenvironment enhances cell migration speed compared to a 2D substrate [69]. In addition, it was found that the cell speed could be reduced by decreasing the pore size of the scaffold since the smaller pore size hindered the cell migration.

As we can note, many scientific papers evaluate the potential of a variety of polymers to reproduce mimetically in vivo environment for organism organization. The results promise to understand the complex cellular mechanisms by cell evaluations in a 3D space with both the large and localized areas.

## 6. Hybrid Ultrafast Laser 3D Processing (Ship-in-a-Bottle Integration)

Hybrid additive and subtractive processing with TPP followed by ultrafast laser multiphoton ablation was successfully demonstrated for fabrication of submicrometer polymer fibers containing periodic holes with 500 nm diameters and 3D microfluidic channels with 1 μm diameters [82]. A “ship-in-a-bottle” fabrication concept based on a hybrid subtractive and additive ultrafast laser processing was introduced in order to integrate 3D polymer structures inside 3D glass micro-channels [83,84].

One example of functional devices developed by the hybrid FLAE-TPP was a multi-functional filter-mixer biochip [83]. It consisted of two filtering sheets placed at the inlet and outlet of a passive type mixer. The mixer presented a configuration of layered crossing tubes to guide and rearrange fluids effectively, so that it could perform fast mixing in a short channel length. This structure integrated by TPP inside a Y-shape closed glass channel demonstrated an excellent mixing performance of two different kinds of fluids with an efficiency of about 87%.

**Figure 9:**
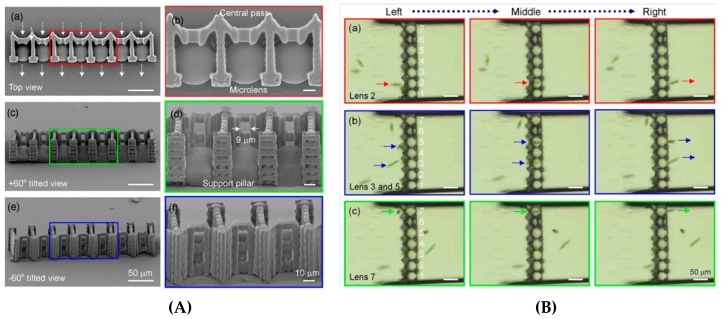
(**A**) TPP fabrication of a center-pass combined microlens array on a flat glass surface. The 9-μm aperture size is slightly larger than the 6–8 μm cells diameter. Fifteen-micrometer-width support pillars were fabricated to increase stability. (**B**) Center-pass function of the optofluidic device to control the Euglena cell position: cell passing above lens 2 and reaching right side of the device (**a**); and cells passing above lenses 3, 5, and 7, from left to right (**b**,**c**). Reproduced from [84].

Another functional biochip fabricated by hybrid FLAE-TPP was an optofluidic platform. The device consisting of 3D microlens array and center pass units was used for *Euglena* cell counting [85]. A 100% success rate in counting was achieved by parallel monitoring of intensity changes induced by *Euglena* cells swimming through center pass units and above microlenses (Figure 9) [84].

**Figure 10:**
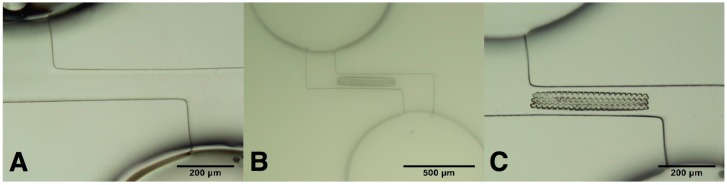
Microscopic images of: (**A**) Z-shaped channel filled with SU-8 photoresist dissolved in acetone and prebaked for 20 h; (**B**) sinusoidal shaped ridges written by TPP, in developing solution; and (**C**) sinusoidal shaped ridges after developing and drying, Reproduced from [86].

Periodic free standing sinusoidal polymeric ridges that can be used for cell manipulation were formed by TPP in channels fabricated by FLAE (Figure 10) [86]. The glass microchannel offered the closed micro-environment, device robustness and dynamic flow simulating conditions for cellular studies while the polymeric integrated patterns reduced the size of structure to the level of which cells are responsive. In addition, the sinusoidal patterns with free-standing stability are exploited, so that they can be used as base scaffolds for building up new structures on their top. Tailoring the periodicity and amplitude spaces between two periodical sinusoidal patterns can host and modulate cell migration. Thus, one foresees some interesting applications for cell manipulation such as guidance and orientation [87]. Since we control cell behavior through mechanical aspects, single cell trapping and analysis are expected to be performed in small areas by using this kind of biochips. Later, hybrid FLAE-TPP approach based on fused silica was also proposed for liposome extrusion, although the integration has not yet been completed [88].

The ultrafast laser processing of both glass and polymer which can create 3D biomimetic environments with resolutions and hierarchy for organisms and cells is at its infancy and has great potential for further development. In addition, integration of optical interrogation components such as microlenses, micro-mirrors as well as optical circuits for performing pump-probe experiments with laser light greatly enhances importance in terms of the technological aspect. It is also of great interest to offer biological platforms fabricated by ultrafast lasers to develop new 3D cell-culture models in order to overcome tissue organization issues arising from conventional analyses using 2D culture systems [89]. Microfluidics approaches will assist to provide the cell-culture microenvironments. It should be noted that current in vitro and animal tests for drug delivery meet problems due to organ complexity and difficulty in predicting drug toxicity [90]. The miniaturized “organs-on-chips” will allow to study human physiology in specific tissues and could replace animal testing for drug development and toxin testing. These novel biochip microdevices take advantage of creation of precisely engineered physiological-like microenvironment and can be combined with well-characterized human cells with small dimensions, which enables high-throughput screening even with small amounts of drugs [91].

## 7. Conclusions and Outlook

Ultrafast laser micro- and nano-technologies such as FLAE, TPP and their hybrid approach have opened up new avenues for research on 3D processing for both transparent inorganic and organic materials. By employing ultrashort pulse widths and extremely high peak intensities, one can implement precise control of the nonlinear interaction region to create complex 3D structures in glass or polymers. For functional biochips, the glass sample offers robustness for easy manipulation and portability, transparency for optical interrogation as well as chemical stability and good biocompatibility. The ultrafast laser induced chemical modification followed by selective etching enables creating 3D microfluidic structures with flexible geometries inside glass. The technique can be extended to the fabrication of microoptics such as micromirrors and microlenses inside a glass substrate. By ultrafast laser processing of photoresists, one can significantly improve the processing resolution with a level of ~100 nm which is beneficial for fabrication of functional microcomponents for biological studies. Thus, TPP has already become a well-known technology for fabricating microdevices with complex designs.

The laser micromachining and micro- and nanostructuring are now accessible to the biomedical field owing to new strategies achieving flexible design and fabrication of biochips. The new hybrid ultrafast laser microfabrication method employs both the 3D microprocessing of glass and 3D “ship-in-a-bottle” polymer integration inside embedded glass channels. Such a hybrid technique proved to combine the specific advantages of individual methods and compensate the drawbacks. The fabricated glass-polymer hybrid microfluidic systems which cover both the scale-down and scale-up aspects can be used as biochips for cell manipulation in 3D environments. The assembled device could be used for monitoring of live cells in dynamic flow conditions. With the aim of controlling and studying cell behavior and their molecular signaling in the most appropriate environment, one could expect that the structural sizes should be further reduced in microfluidic devices while the sensitivity of analytic procedures should be increased. The sizes of structures can be reduced to the level of single cell dimensions at which cells can be influenced and manipulated inside glass microchannel. Furthermore, due to fabrication of biomimetic micro- and nano-environments, it is highly expected that new miniaturized organs will be developed that can provide similar in vivo conditions.

## Figures and Tables

**Table 1:** Ultrafast laser 3D processing methods for biochip fabrication: advantages and drawbacks.

Ultrafast Laser Processing Method	Advantages	Drawbacks
FLAE (FLICE) of glasses	Large area processing; Robustness; Portability; Transparency; Biocompatibility	Low resolution processing; Low flexibility; Type of glass limited
TPP of polymers	High resolution processing; Elasticity; Transparency; Biocompatibility	Small area processing; Fragile structure

## References

[1] Chichkov B.N., Momma C., Nolte S., Von Alvensleben F., Tünnermann A. (1996). Femtosecond, picosecond and nanosecond laser ablation of solids. Appl. Phys. A.

[2] Schaffer C.B., Brodeur A., Mazur E. (2001). Laser-induced breakdown and damage in bulk transparent materials induced by tightly focused femtosecond laser pulses. Meas. Sci. Technol..

[3] Gattass R.R., Mazur E. (2008). Femtosecond laser micromachining in transparent materials. Nat. Photonics.

[4] Du D., Liu X., Korn G., Squier J., Mourou G. (1994). Laser-induced breakdown by impact ionization in sio2 with pulse widths from 7 ns to 150 fs. Appl. Phys. Lett..

[5] Pronko P., Dutta S., Squier J., Rudd J., Du D., Mourou G. (1995). Machining of sub-micron holes using a femtosecond laser at 800 nm. Opt. Commun..

[6] Krüger J., Kautek W. (1996). Femtosecond-pulse visible laser processing of transparent materials. Appl. Surf. Sci..

[7] Schaffer C.B., Brodeur A., García J.F., Mazur E. (2001). Micromachining bulk glass by use of femtosecond laser pulses with nanojoule energy. Opt. Lett..

[8] Sakakura M., Terazima M. (2004). Oscillation of the refractive index at the focal region of a femtosecond laser pulse inside a glass. Opt. Lett..

[9] Sakakura M., Terazima M., Shimotsuma Y., Miura K., Hirao K. (2007). Observation of pressure wave generated by focusing a femtosecond laser pulse inside a glass. Opt. Express.

[10] Davis K.M., Miura K., Sugimoto N., Hirao K. (1996). Writing waveguides in glass with a femtosecond laser. Opt. Lett..

[11] Kondo Y., Qiu J., Mitsuyu T., Hirao K., Yoko T. (1999). Three-dimensional microdrilling of glass by multiphoton process and chemical etching. Jpn. J. Appl. Phys..

[12] Marcinkevičius A., Juodkazis S., Watanabe M., Miwa M., Matsuo S., Misawa H., Nishii J. (2001). Femtosecond laser-assisted three-dimensional microfabrication in silica. Opt. Lett..

[13] Masuda M., Sugioka K., Cheng Y., Aoki N., Kawachi M., Shihoyama K., Toyoda K., Helvajian H., Midorikawa K. (2003). 3-D microstructuring inside photosensitive glass by femtosecond laser excitation. Appl. Phys. A.

[14] Watanabe W., Sowa S., Tamaki T., Itoh K., Nishii J. (2006). Three-dimensional waveguides fabricated in poly (methyl methacrylate) by a femtosecond laser. Jpn. J. Appl. Phys..

[15] Suriano R., Kuznetsov A., Eaton S.M., Kiyan R., Cerullo G., Osellame R., Chichkov B.N., Levi M., Turri S. (2011). Femtosecond laser ablation of polymeric substrates for the fabrication of microfluidic channels. Appl. Surf. Sci..

[16] De Marco C., Eaton S.M., Suriano R., Turri S., Levi M., Ramponi R., Cerullo G., Osellame R. (2010). Surface properties of femtosecond laser ablated pmma. ACS Appl. Mater. Interfaces.

[17] Sowa S., Watanabe W., Tamaki T., Nishii J., Itoh K. (2006). Symmetric waveguides in poly (methyl methacrylate) fabricated by femtosecond laser pulses. Opt. Express.

[18] Baudach S., Bonse J., Krüger J., Kautek W. (2000). Ultrashort pulse laser ablation of polycarbonate and polymethylmethacrylate. Appl. Surf. Sci..

[19] Kallepalli D.L., Desai N.R., Soma V.R. (2010). Fabrication and optical characterization of microstructures in poly (methylmethacrylate) and poly (dimethylsiloxane) using femtosecond pulses for photonic and microfluidic applications. Appl. Opt..

[20] Yong J., Chen F., Yang Q., Du G., Bian H., Zhang D., Si J., Yun F., Hou X. (2013). Rapid fabrication of large-area concave microlens arrays on pdms by a femtosecond laser. ACS Appl. Mater. Interfaces.

[21] Sun H.-B., Kawata S. (2004). Two-photon photopolymerization and 3D lithographic microfabrication. NMR • 3D Analysis • Photopolymerization.

[22] Park S.H., Yang D.Y., Lee K.S. (2009). Two-photon stereolithography for realizing ultraprecise three-dimensional nano/microdevices. Laser Photonics Rev..

[23] Zhang Y.-L., Chen Q.-D., Xia H., Sun H.-B. (2010). Designable 3D nanofabrication by femtosecond laser direct writing. Nano Today.

[24] Stratakis E., Ranella A., Farsari M., Fotakis C. (2009). Laser-based micro/nanoengineering for biological applications. Prog. Quantum Electron..

[25] Itoh K., Watanabe W., Nolte S., Schaffer C.B. (2006). Ultrafast processes for bulk modification of transparent materials. MRS Bull..

[26] Sugioka K., Cheng Y. (2012). Femtosecond laser processing for optofluidic fabrication. Lab Chip.

[27] Osellame R., Hoekstra H.J., Cerullo G., Pollnau M. (2011). Femtosecond laser microstructuring: An enabling tool for optofluidic lab-on-chips. Laser Photonics Rev..

[28] Sugioka K., Cheng Y. (2014). Femtosecond laser three-dimensional micro-and nanofabrication. Appl. Phys. Rev..

[29] Sugioka K., Xu J., Wu D., Hanada Y., Wang Z., Cheng Y., Midorikawa K. (2014). Femtosecond laser 3D micromachining: A powerful tool for the fabrication of microfluidic, optofluidic, and electrofluidic devices based on glass. Lab Chip.

[30] Sugioka K., Cheng Y. (2014). Ultrafast lasers—Reliable tools for advanced materials processing. Light Sci. Appl..

[31] Della Valle G., Osellame R., Laporta P. (2008). Micromachining of photonic devices by femtosecond laser pulses. J. Opt. A: Pure Appl. Opt..

[32] Stratakis E., Ranella A., Fotakis C. (2011). Biomimetic micro/nanostructured functional surfaces for microfluidic and tissue engineering applications. Biomicrofluidics.

[33] Schmidt V., Belegratis M. (2013). Laser Technology in Biomimetics.

[34] Manz A., Harrison D.J., Verpoorte E.M., Fettinger J.C., Paulus A., Lüdi H., Widmer H.M. (1992). Planar chips technology for miniaturization and integration of separation techniques into monitoring systems: Capillary electrophoresis on a chip. J. Chromatogr. A.

[35] Harrison D.J., Fluri K., Seiler K., Fan Z., Effenhauser C.S., Manz A. (1993). Micromachining a miniaturized capillary electrophoresis-based chemical analysis system on a chip. Science.

[36] Becker H., Locascio L.E. (2002). Polymer microfluidic devices. Talanta.

[37] Heckele M., Schomburg W. (2003). Review on micro molding of thermoplastic polymers. J. Micromech. Microeng..

[38] Gower M.C. (2000). Industrial applications of laser micromachining. Opt. Express.

[39] Chrisey D., Pique A., McGill R., Horwitz J., Ringeisen B., Bubb D., Wu P. (2003). Laser deposition of polymer and biomaterial films. Chem. Rev..

[40] Barbulovic-Nad I., Lucente M., Sun Y., Zhang M., Wheeler A.R., Bussmann M. (2006). Bio-microarray fabrication techniques—A review. Crit. Rev. Biotechnol..

[41] Ilina O., Bakker G.-J., Vasaturo A., Hoffman R.M., Friedl P. (2011). Two-photon laser-generated microtracks in 3D collagen lattices: Principles of mmp-dependent and-independent collective cancer cell invasion. Phys. Biol..

[42] Moriguchi H., Takahashi K., Sugio Y., Wakamoto Y., Inoue I., Jimbo Y., Yasuda K. (2004). On-chip neural cell cultivation using agarose-microchamber array constructed by a photothermal etching method. Electr. Eng. Jpn..

[43] Barbucci R., Lamponi S., Pasqui D., Rossi A., Weber E. (2003). Micropatterned polysaccharide surfaces via laser ablation for cell guidance. Mater. Sci. Eng. C.

[44] Bellouard Y., Said A., Dugan M., Bado P. (2004). Fabrication of high-aspect ratio, micro-fluidic channels and tunnels using femtosecond laser pulses and chemical etching. Opt. Express.

[45] Vishnubhatla K.C., Bellini N., Ramponi R., Cerullo G., Osellame R. (2009). Shape control of microchannels fabricated in fused silica by femtosecond laser irradiation and chemical etching. Opt. Express.

[46] He S., Chen F., Yang Q., Liu K., Shan C., Bian H., Liu H., Meng X., Si J., Zhao Y. (2012). Facile fabrication of true three-dimensional microcoils inside fused silica by a femtosecond laser. J. Micromech. Microeng..

[47] Yamada K., Watanabe W., Toma T., Itoh K., Nishii J. (2001). In situ observation of photoinduced refractive-index changes in filaments formed in glasses by femtosecond laser pulses. Opt. Lett..

[48] Hongo T., Sugioka K., Niino H., Cheng Y., Masuda M., Miyamoto I., Takai H., Midorikawa K. (2005). Investigation of photoreaction mechanism of photosensitive glass by femtosecond laser. J. Appl. Phys..

[49] Cheng Y., Tsai H.-L., Sugioka K., Midorikawa K. (2006). Fabrication of 3D microoptical lenses in photosensitive glass using femtosecond laser micromachining. Appl. Phys. A.

[50] Hanada Y., Sugioka K., Shihira-Ishikawa I., Kawano H., Miyawaki A., Midorikawa K. (2011). 3D microfluidic chips with integrated functional microelements fabricated by a femtosecond laser for studying the gliding mechanism of cyanobacteria. Lab Chip.

[51] Hanada Y., Sugioka K., Midorikawa K. (2012). Highly sensitive optofluidic chips for biochemical liquid assay fabricated by 3D femtosecond laser micromachining followed by polymer coating. Lab Chip.

[52] Wang Z., Sugioka K., Midorikawa K. (2008). Fabrication of integrated microchip for optical sensing byáfemtosecond laser direct writing of foturan glass. Appl. Phys. A.

[53] Crespi A., Gu Y., Ngamsom B., Hoekstra H.J., Dongre C., Pollnau M., Ramponi R., van den Vlekkert H.H., Watts P., Cerullo G. (2010). Three-dimensional mach-zehnder interferometer in a microfluidic chip for spatially-resolved label-free detection. Lab Chip.

[54] Hanada Y., Sugioka K., Kawano H., Ishikawa I.S., Miyawaki A., Midorikawa K. (2008). Nano-aquarium for dynamic observation of living cells fabricated by femtosecond laser direct writing of photostructurable glass. Biomed. Microdevices.

[55] Hanada Y., Sugioka K., Kawano H., Ishikawa I.S., Miyawaki A., Midorikawa K. (2009). Nano-aquarium with microfluidic structures for dynamic analysis of cryptomonas and phormidium fabricated by femtosecond laser direct writing of photostructurable glass. Appl. Surf. Sci..

[56] Xu J., Wu D., Hanada Y., Chen C., Wu S., Cheng Y., Sugioka K., Midorikawa K. (2013). Electrofluidics fabricated by space-selective metallization in glass microfluidic structures using femtosecond laser direct writing. Lab Chip.

[57] Xu J., Wu D., Ip J.Y., Midorikawa K., Sugioka K. (2015). Vertical sidewall electrodes monolithically integrated into 3D glass microfluidic chips using water-assisted femtosecond-laser fabrication for in situ control of electrotaxis. RSC Adv..

[58] Kiyama S., Matsuo S., Hashimoto S., Morihira Y. (2009). Examination of etching agent and etching mechanism on femotosecond laser microfabrication of channels inside vitreous silica substrates^†^. J. Phys. Chem. C.

[59] Maselli V., Osellame R., Cerullo G., Ramponi R., Laporta P., Magagnin L., Cavallotti P.L. (2006). Fabrication of long microchannels with circular cross section using astigmatically shaped femtosecond laser pulses and chemical etching. Appl. Phys. Lett..

[60] Hwang D.J., Kim M., Hiromatsu K., Jeon H., Grigoropoulos C.P. (2009). Three-dimensional opto-fluidic devices fabricated by ultrashort laser pulses for high throughput single cell detection and processing. Appl. Phys. A.

[61] Bragheri F., Ferrara L., Bellini N., Vishnubhatla K.C., Minzioni P., Ramponi R., Osellame R., Cristiani I. (2010). Optofluidic chip for single cell trapping and stretching fabricated by a femtosecond laser. J. Biophotonics.

[62] Yang T., Paiè P., Nava G., Bragheri F., Vazquez R.M., Minzioni P., Veglione M., Di Tano M., Mondello C., Osellame R. (2015). An integrated optofluidic device for single-cell sorting driven by mechanical properties. Lab Chip.

[63] Bragheri F., Minzioni P., Vazquez R.M., Bellini N., Paie P., Mondello C., Ramponi R., Cristiani I., Osellame R. (2012). Optofluidic integrated cell sorter fabricated by femtosecond lasers. Lab Chip.

[64] Tanaka T., Sun H.-B., Kawata S. (2002). Rapid sub-diffraction-limit laser micro/nanoprocessing in a threshold material system. Appl. Phys. Lett..

[65] Sun H.-B., Matsuo S., Misawa H. (1999). Three-dimensional photonic crystal structures achieved with two-photon-absorption photopolymerization of resin. Appl. Phys. Lett..

[66] Wu D., Chen Q.-D., Niu L.-G., Wang J.-N., Wang J., Wang R., Xia H., Sun H.-B. (2009). Femtosecond laser rapid prototyping of nanoshells and suspending components towards microfluidic devices. Lab Chip.

[67] Wang J., He Y., Xia H., Niu L.-G., Zhang R., Chen Q.-D., Zhang Y.-L., Li Y.-F., Zeng S.-J., Qin J.-H. (2010). Embellishment of microfluidic devices via femtosecond laser micronanofabrication for chip functionalization. Lab Chip.

[68] Amato L., Gu Y., Bellini N., Eaton S.M., Cerullo G., Osellame R. (2012). Integrated three-dimensional filter separates nanoscale from microscale elements in a microfluidic chip. Lab Chip.

[69] Tayalia P., Mendonca C.R., Baldacchini T., Mooney D.J., Mazur E. (2008). 3D cell-migration studies using two-photon engineered polymer scaffolds. Adv. Mater..

[70] Ovsianikov A., Schlie S., Ngezahayo A., Haverich A., Chichkov B.N. (2007). Two-photon polymerization technique for microfabrication of cad-designed 3D scaffolds from commercially available photosensitive materials. J. Tissue Eng. Regener. Med..

[71] Ovsianikov A., Malinauskas M., Schlie S., Chichkov B., Gittard S., Narayan R., Löbler M., Sternberg K., Schmitz K.-P., Haverich A. (2011). Three-dimensional laser micro-and nano-structuring of acrylated poly (ethylene glycol) materials and evaluation of their cytoxicity for tissue engineering applications. Acta Biomater..

[72] Hidai H., Jeon H., Hwang D.J., Grigoropoulos C.P. (2009). Self-standing aligned fiber scaffold fabrication by two photon photopolymerization. Biomed. Microdevices.

[73] Sima L., Buruiana E., Buruiana T., Matei A., Epurescu G., Zamfirescu M., Moldovan A., Petrescu S., Dinescu M. (2013). Dermal cells distribution on laser-structured ormosils. J. Tissue Eng. Regener. Med..

[74] Raimondi M.T., Eaton S.M., Nava M.M., Laganà M., Cerullo G., Osellame R. (2012). Two-photon laser polymerization: From fundamentals to biomedical application in tissue engineering and regenerative medicine. J. Appl. Biomater. Funct. Mater..

[75] Klein F., Richter B., Striebel T., Franz C.M., Freymann G.V., Wegener M., Bastmeyer M. (2011). Two-component polymer scaffolds for controlled three-dimensional cell culture. Adv. Mater..

[76] Fadeeva E., Deiwick A., Chichkov B., Schlie-Wolter S. (2014). Impact of laser-structured biomaterial interfaces on guided cell responses. Interface Focus.

[77] Marino A., Filippeschi C., Mattoli V., Mazzolai B., Ciofani G. (2015). Biomimicry at the nanoscale: Current research and perspectives of two-photon polymerization. Nanoscale.

[78] Kim M., Hwang D.J., Jeon H., Hiromatsu K., Grigoropoulos C.P. (2009). Single cell detection using a glass-based optofluidic device fabricated by femtosecond laser pulses. Lab Chip.

[79] Raimondi M.T., Eaton S.M., Laganà M., Aprile V., Nava M.M., Cerullo G., Osellame R. (2013). Three-dimensional structural niches engineered via two-photon laser polymerization promote stem cell homing. Acta Biomater..

[80] Klein F., Striebel T., Fischer J., Jiang Z., Franz C.M., von Freymann G., Wegener M., Bastmeyer M. (2010). Elastic fully three-dimensional microstructure scaffolds for cell force measurements. Adv. Mater..

[81] Thiery J.P., Acloque H., Huang R.Y., Nieto M.A. (2009). Epithelial-mesenchymal transitions in development and disease. Cell.

[82] Xiong W., Zhou Y.S., He X.N., Gao Y., Mahjouri-Samani M., Jiang L., Baldacchini T., Lu Y.F. (2012). Simultaneous additive and subtractive three-dimensional nanofabrication using integrated two-photon polymerization and multiphoton ablation. Light: Sci. Appl..

[83] Wu D., Wu S.Z., Xu J., Niu L.G., Midorikawa K., Sugioka K. (2014). Hybrid femtosecond laser microfabrication to achieve true 3D glass/polymer composite biochips with multiscale features and high performance: The concept of ship-in-a-bottle biochip. Laser Photonics Rev..

[84] Wu D., Niu L.-G., Wu S.-Z., Xu J., Midorikawa K., Sugioka K. (2015). Ship-in-a-bottle femtosecond laser integration of optofluidic microlens arrays with center-pass units enabling coupling-free parallel cell counting with a 100% success rate. Lab Chip.

[85] Wu D., Xu J., Niu L.-G., Wu S.-Z., Midorikawa K., Sugioka K. (2015). In-channel integration of designable microoptical devices using flat scaffold-supported femtosecond-laser microfabrication for coupling-free optofluidic cell counting. Light: Sci. Appl..

[86] Sima F., Wu D., Xu J., Midorikawa K., Sugioka K. (2015). Ship-in-a-bottle integration by hybrid femtosecond laser technology for fabrication of true 3D biochips. Proc. SPIE.

[87] Gamboa J.R., Mohandes S., Tran P.L., Slepian M.J., Yoon J.-Y. (2013). Linear fibroblast alignment on sinusoidal wave micropatterns. Colloids Surf. B: Biointerfaces.

[88] Baldacchini T., Nuñez V., LaFratta C.N., Grech J.S., Vullev V.I., Zadoyan R. (2015). Microfabrication of three-dimensional filters for liposome extrusion. Proc. SPIE.

[89] Huh D., Hamilton G.A., Ingber D.E. (2011). From 3D cell culture to organs-on-chips. Trends Cell Biol..

[90] Beebe D.J., Ingber D.E., den Toonder J. (2013). Organs on chips 2013. Lab Chip.

[91] Beißner N., Lorenz T., Reichl S. (2016). Organ on chip. Microsystems for Pharmatechnology.

